# Implementing a comprehensive translational oncology platform: from molecular testing to actionability

**DOI:** 10.1186/s12967-018-1733-y

**Published:** 2018-12-14

**Authors:** Zahi I. Mitri, Swapnil Parmar, Brett Johnson, Annette Kolodzie, Jamie M. Keck, Max Morris, Alexander R. Guimaraes, Brooke R. Beckett, Uma Borate, Charles D. Lopez, Kathleen A. Kemmer, Joshi J. Alumkal, Tomasz M. Beer, Christopher L. Corless, Gordon B. Mills, Joe W. Gray, Raymond C. Bergan

**Affiliations:** Oregon Health and Science University, 3181 SW Sam Jackson Hall, Portland, OR 97239-3098 USA

**Keywords:** Precision oncology, Translational research, Targeted therapy, Biomarkers, Clinical trials

## Abstract

**Background:**

In order to establish the workflows required to implement a real-time process involving multi-omic analysis of patient samples to support precision-guided therapeutic intervention, a tissue acquisition and analysis trial was implemented. This report describes our findings to date, including the frequency with which mutational testing led to precision-guided therapy and outcome for those patients.

**Methods:**

Eligible patients presenting to Oregon Health and Science University Knight Cancer Institute were enrolled on the study. Patients with biopsy proven metastatic or locally advanced unresectable prostate cancer, breast cancer, pancreatic adenocarcinoma, or refractory acute myelogenous leukemia receiving standard of care therapy were eligible. Metastatic site biopsies were collected and analyzed using the Knight Diagnostic Lab GeneTrails comprehensive solid tumor panel (124 genes). CLIA certified genomic information was made available to the treating physician.

**Results:**

Between 1/26/2017 and 5/30/2018, 38 patients were enrolled, with 28 successfully undergoing biopsy. Of these, 25 samples yielded sufficient tumor for analysis. The median biopsy cellularity and number of cores collected were 70% (15–90%) and 5 (2–20), respectively. No procedure-related complications occurred. GeneTrails analysis revealed that 22 of 25 (88%) tumor samples harbored at least one potentially actionable mutation, and 18 (72%) samples harbored 2 or more potentially actionable mutations. The most common genetic alterations identified involved: DNA damage repair genes, cell cycle regulating genes, *PIK3CA/Akt/mTOR* pathway, and FGF gene family. To date, CLIA certified genomic results were used by treating physicians for precision-guided therapy in 5 (23%) patients.

**Conclusion:**

We report the feasibility of real-time tissue acquisition and analysis to support a successful translational oncology platform. The workflow will provide the foundation to improve access and accrual to biomarker driven precision oncology trials.

**Electronic supplementary material:**

The online version of this article (10.1186/s12967-018-1733-y) contains supplementary material, which is available to authorized users.

## Background

Rapid advances in technology have revolutionized our understanding of cancer biology. Next generation sequencing methods have allowed us to map the genetic landscape for different tumor types [1–3]. This information has paved the way for precision medicine, wherein therapy is chosen to interdict specific molecular defects present in a patient’s tumor with the goal of controlling cancer, limiting toxicity and improving patient outcomes. The implementation of precision medicine has changed treatment paradigms in several settings, including EGFR and ALK inhibitors in non-small cell lung cancer [4–6], BRAF inhibitors in malignant melanoma [7], BCR-ABL inhibitors in chronic myelogenous leukemia [8], and PARP inhibitors in breast and ovarian cancers [9–11], among several others [12]. As a result–and because of increased demand from patients and physicians alike, tumor genomic sequencing platforms are increasingly being used to guide standard of care therapy as well as enrollment on clinical trials.

Unfortunately, leveraging tumor molecular profiling to deliver on the promise of precision medicine has been faced with several challenges, with only a minority of tested patients receiving genotype-matched targeted therapies in clinical trials [13]. These challenges have included institutional factors such as clinical trial availability, patient factors such as eligibility criteria, availability of adequate tissue for testing, identifying actionable or druggable alterations, and laboratory factors such as lack of standardization among different sequencing platforms, and prolonged reporting times [14]. Published experiences from large centers have reflected this reality, with less than 10% of patients receiving therapy on genotype-matched trials, despite 30% or more having potentially targetable genetic alterations [15, 16]. Additionally, there are conflicting reports as to the survival benefit of treatment based on identified actionable DNA mutations compared to standard of care therapy [17, 18]. It is important to emphasize as indicated above that multiple targeted therapy approaches are now considered standard of care and thus are not included as part of clinical trial designs to test the utility of the platform. Indeed, using enrollment on clinical trials as a readout of utility greatly underestimates the impact of genetic testing and precision oncology efforts.

With a clear knowledge of the current landscape, the Oregon Health and Science University Knight Cancer Institute (OHSU KCI) set out to implement an innovative precision oncology platform. The conceptual underpinnings of that platform are: cancer is a highly adaptive state driven by more than a one biological process, there is much more to understanding cancer biology than that garnered solely from DNA-based analytics, and information from a broader set of analytics conducted at the individual patient level has a high potential to inform therapeutic decisions. The corollary to this is that monotherapy has inherent limitations that combination therapies targeting multiple pathways simultaneously may address. From this conceptual basis, our group designed and is launching the Serial Measurements of Molecular and Architectural Responses to Therapy (SMMART) trials initiative. In broad terms, the SMMART initiative will conduct a multi-omic analysis on tumors from patients with refractory cancer and will use that information to guide and deliver targeted therapy.

To achieve this aim, a comprehensive set of analytics will be applied to tumor samples, including: whole exome sequencing of tumor and circulating DNA, RNA transcriptional profiling, protein expression analysis, multiplexed spatially resolved immunohistochemical and immunofluorescent staining, and functional profiling of drug sensitivity of metastatic tumors grown in culture. Recognizing that tissue samples are inherently small in quantity and that proposed analytics will need adequate tissue to support them, an initial goal of the SMMART program is to develop the workflow involved in tissue acquisition and comprehensive multi-omic analysis, specifically to establish a set of standard operating procedures that can be conducted in real-time to effectively guide treatment for patients with advanced cancer. This workflow will be initially evaluated in the research environment followed by rapid transition of approaches with high information content to the CLIA laboratory. This report describes metrics associated with conducting biopsies on patients with advanced cancer, including: biopsy success rates, analytical success rates, turnaround times of genomic analyses reports, and impact of testing on treatment decisions and patient outcomes.

## Methods

### Patients

Patients presenting to OHSU KCI with biopsy-proven metastatic or locally advanced unresectable prostate cancer, breast cancer, pancreatic adenocarcinoma and refractory acute myelogenous leukemia were eligible for the study. Patients were enrolled under the IRB approved “Molecular Mechanisms of Tumor Evolution and Resistance to Therapy” (MM-TERT) clinical protocol. SMMART program research coordinators were responsible for patient consent, arranging tissue sampling and processing, ensuring timely reporting of CLIA assays to study scientists and treating physicians, and finally follow up patients for impact of MM-TERT assays on treatment decisions and patient clinical outcomes. Eligibility criteria included: ECOG PS 0-2, metastatic disease amenable to biopsy, and lack of bleeding diatheses. We report here MM-TERT patients enrolled between 1/26/2017 and 5/30/2018.

### Biopsy methods

Patients deemed eligible for consideration of biopsy had their imaging reviewed by experienced diagnostic radiologists with expertise in body and musculoskeletal imaging to estimate biopsy feasibility and safety. Prior to consent, review of significant co-morbidities which may interfere with optimal positioning, anti-coagulant or anti-platelet medication use, review of complete blood counts and coagulation test results, allergies and ability to comply was completed. The lesion was identified by either ultrasound or CT imaging, and a safe trajectory was identified for biopsy. Patients received sedation per routine clinical protocol. This was followed by percutaneous insertion of a 17 gauge metal introducer into the lesion with visual confirmation by either CT or ultrasound. Two to seven 2 cm long 18-gauge core biopsy tissue samples were obtained using a cutting core biopsy needle and submitted for analysis. Post biopsy images were obtained in order to confirm absence of complications or adverse events.

### Genomic testing

All testing was performed in a CLIA-licensed, CAP-certified laboratory (Knight Diagnostic Laboratories (KDL), OHSU).

Unstained sections of formalin-fixed paraffin-embedded tumor tissue were reviewed by a board-certified pathologist and areas of tumor were enriched by macrodissection. Total nucleic acid was extracted and purified using a commercially available kit (Machery-Nagel). Twenty nanograms of DNA was sequenced using a custom 124 cancer gene panel (GeneTrails^®^ Comprehensive Solid Tumor Panel). The genes represented on this panel are considered potentially clinically actionable and were selected based on their relevance to current clinical therapies (Additional file 1: Table S1). Of the 124 genes, all coding regions are sequenced for 103 genes; known regions of mutation were covered for the remaining 22 genes. The panel is based on a custom AmpliSeq primer set (5297 amplicons; ThermoFisher) and is run on an Illumina NextSeq 500 (2 × 150 bp), at an average read depth > 2000 for each gene. GATK tools (Broad Institute) are used for sequence alignment and variant calling. A custom script is used for copy number estimation [19]. Normal DNA from the patients was not sequenced, so the germline status of identified mutations was not assessed.

Microsatellite instability was measured using a commercially available kit (Promega) that measures 5 standard mononucleotide repeat markers (BAT-25, BAT-26, NR-21, NR-24 and MONO-27) through a combination of PCR and capillary electrophoresis.

Gene fusions were screened using an RNA-sequencing assay (GeneTrails^®^ Gene Fusion Panel) that is based on a custom QIAseq amplicon library (Qiagen). This assay can detect fusions involving AKT3, ALK, BRAF, EFGR, EML4, ERBB4, ERG, ETV6, FGFR1, FGFR2, FGFR3, MET, NOTCH1, NOTCH2, NRG1, NTRK1, NTRK2, NTRK3, NUTM1, PDGFRA, RAF1, and RET. The assay is designed to detect any fusion partner involving these genes. The panel requires an input of 30 ng of RNA and is run on a NextSeq 500. A minimum of 100,000 unique, mapped reads is required for analysis. Following sequence alignment (GATK tools), fusions are detected using StarFusion.

### Immunohistochemistry

Immunohistochemical staining was performed in the Department of Pathology using commercial kits (Ventana, Roche Diagnostics) for the following markers: ER, PR, AR, HER2, Ki-67. Immunostaing for PD-L1 (pharmDx clone 22C3) was performed by PhenoPath Laboratories (Seattle, WA). Immunostains for p16, RB, PTEN, phospho-AKT and phospho-ERK were performed in the laboratory of Dr. Robert Brown, University of Texas, Houston.

### Mutation designation

Clinically actionable mutations were defined as genetic alterations that potentially can be targetable with investigational agents. Alterations in *TP53* and *KRAS* genes were not considered actionable.

### Clinical patient data

Electronic medical records were reviewed for patient demographics, tumor type and stage, sites of metastatic disease and treatment history. Biopsy date, site, adverse events, and tumor cellularity information were collected.

## Results

### Patient population

**Fig. 1:**
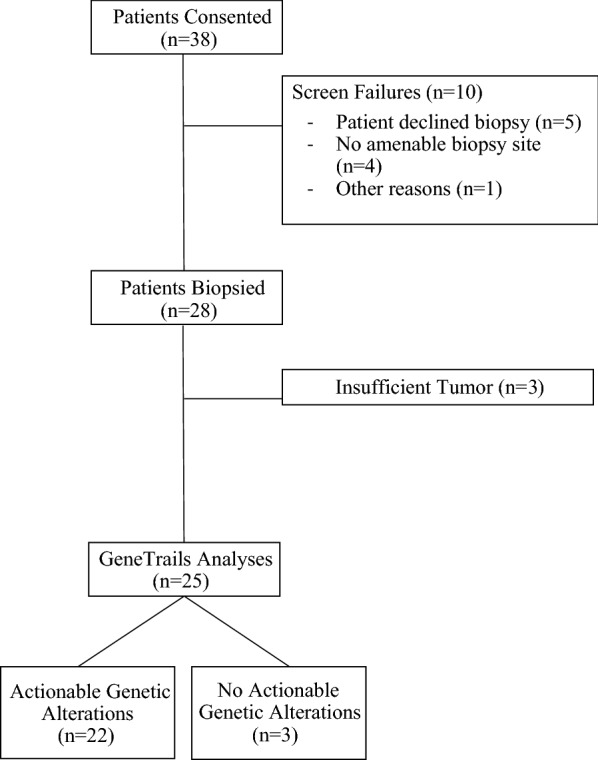
MM-TERT study consort flow diagram

Between 1/26/2017 and 5/30/2018, 38 patients were enrolled on MM-TERT and 28 underwent tumor biopsy (Fig. 1). All patients had biopsy-proven metastatic disease, and were either undergoing or planning to start therapy. Patient demographics are summarized in Table 1. Of the four cancer types potentially eligible, n = 19 had breast cancer, n = 18 had prostate cancer and n = 1 had pancreatic cancer. This reflected the availability of established on-site programs for pancreatic cancer and AML, and led to low enrollment of these patients on this study.

**Table 1:** Patient demographics

	Breast cancer (n = 19)	Prostate cancer (n = 18)	Pancreatic cancer (n = 1)	Total (n = 38)
Gender				
Male	0	18	1	19
Female	19	0	0	19
Age in years (median, range)	56 (35–71)	68 (56–80)	82	65 (35–82)
Prior therapies (median, range)	1.5 (0–14)	4 (0–9)	1	3 (0–14)
ECOG PS				
0–1	16	18	1	31
≥ 2	3	0	0	3
Research biopsy performed	15	12	1	28
Biopsy cellularity % (median, range)	70 (15–80)	75 (50–90)	80	70 (15–90)
Number of cores (median, range)	5 (3–10)	4.5 (2–7)	20	6 (2–20)

### Procedures

Among 38 consented patients, 28 patients (71%) successfully underwent protocol mandated biopsies. There were 10 screen failures related to patient declining biopsy (n = 5), lack of sites amenable to biopsy (n = 4), or other unspecified reasons (n = 1).

Biopsy sites included liver, bone, skin, soft tissue, and lymph node metastases. No biopsy related adverse events occurred. Median time from consent to biopsy was 9 days (range 0–49 days). It should be noted that in some instances biopsy was synchronized with the next scheduled visit, so as to diminish patient burden. Median biopsy cellularity was 70%, and median number of cores obtained per biopsy was 5 (Table 1).

### Genomic analyses

GeneTrails was performed on 25 of the 28 collected research biopsies. Three patient biopsies had insufficient tumor for analyses. Of those, one breast cancer patient subsequently achieved complete radiographic response on restaging scans, and one prostate cancer patient had sclerotic tissue on bone biopsy, which is a determinant of biopsy yield [20]. Another prostate cancer patient’s bone biopsy yielded normal tissue. Median time from biopsy date to GeneTrails report was 17 days (range 11–46 days).

**Fig. 2:**
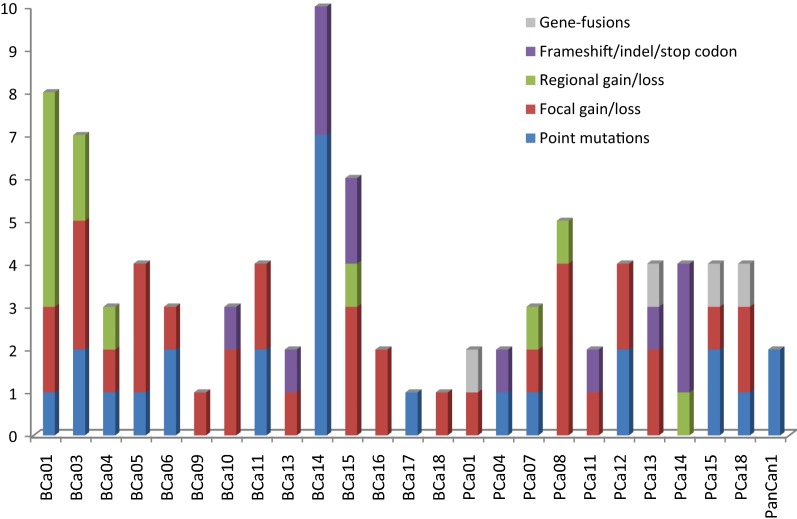
Type of genetic alterations identified by genetrails analysis for each patient. *BCa* breast cancer, *PCa* prostate cancer, *PanCa* pancreatic cancer

Of the 25 evaluable patient tumors, 22 (88%) harbored at least one potentially actionable genetic alteration. Notably, 18 tumors were found to have 2 or more potentially actionable genetic alterations. Of 25 evaluable tumors, genetic alterations identified included: 21 copy number alterations (84%; breast and prostate), 14 point mutations (56%; breast, prostate and pancreas), 8 structural mutations (32%; breast and prostate) and 4 gene fusions (16%; prostate) (Fig. 2, Additional file 1: Table S1).

**Fig. 3:**
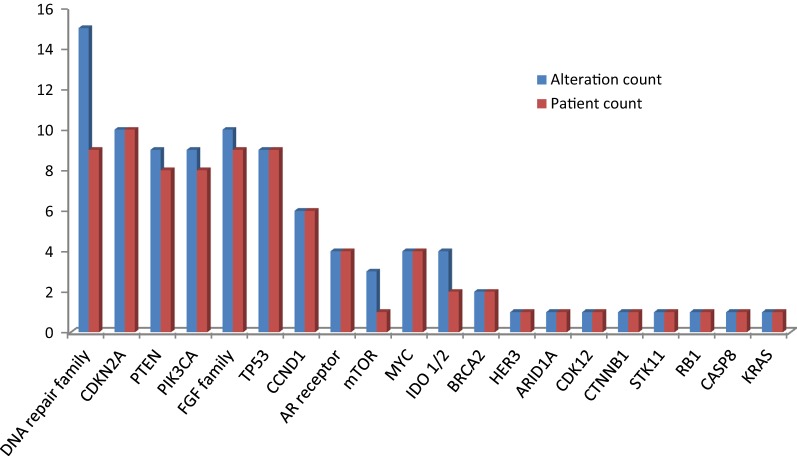
Frequency of genetic alterations identified by GeneTrails analysis

Of 25 evaluable tumors, the most common genomic pathways involved included cell cycle regulating genes such as *CDKN2A* 10/25 (40%), and *CCND1* 6/25 (24%), DNA damage repair genes 9/25 (36%);; the *PIK3CA/Akt/mTOR* pathway such as *PTEN* (8/25, 32%), and *PIK3CA* (8/25, 32%); and *FGFR* alterations (9/25, 36%). Of note, *TP53* alterations were identified in 8 cases (32%), and *KRAS* alteration was reported in the patient with pancreatic adenocarcinoma (Fig. 3). Considering the small sample size, the frequencies were in line with expected frequency of aberrations.

#### Breast cancer cohort

**Fig. 4:**
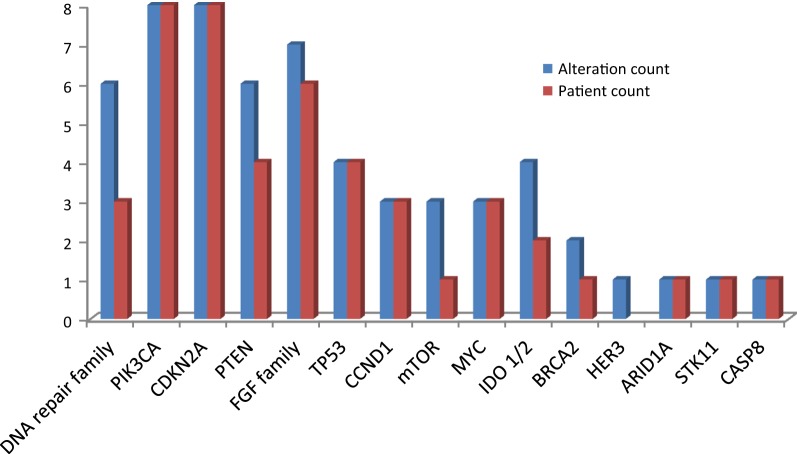
Frequency of genetic alterations identified by GeneTrails analysis in the breast cancer cohort

Among the 14 evaluable breast cancer patients who underwent a research biopsy, 9 had hormone receptor-positive disease, 1 had HER2-amplified disease, 1 had HER2 status change from positive to negative, and 2 patients had triple negative breast cancer. The most common actionable genetic alterations in the breast cancer cohort included: 8 *PIK3CA* (57%), 8 *CDKN2A* (57%), 6 *FGFR* (42%), 3 patients with DNA damage repair alterations (21%), and 4 *PTEN* (28%) (Fig. 4).

#### Prostate cancer cohort

**Fig. 5:**
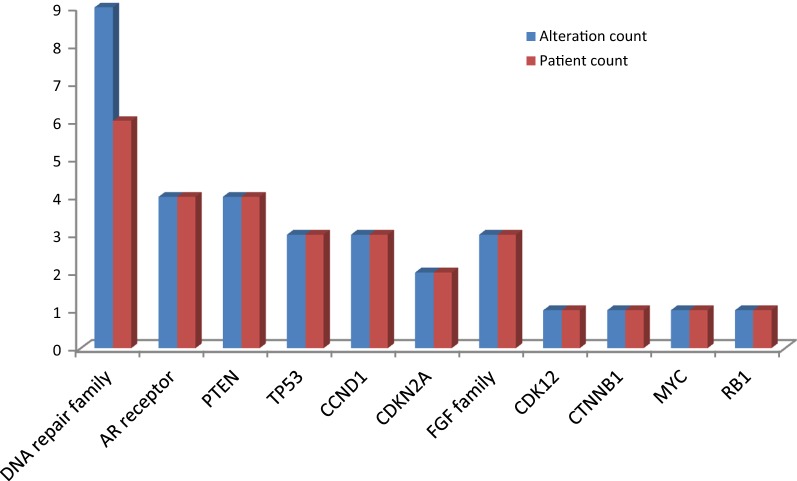
Frequency of genetic alterations identified by GeneTrails analysis in the prostate cancer cohort

Among the ten evaluable prostate cancer patients who underwent a research biopsy, 9 had metastatic castrate-resistant prostate cancer and 1 patient had castration-sensitive prostate cancer. All patients had prostate adenocarcinoma. The most common actionable genetic alterations in the prostate cancer cohort included: 6 DNA damage repair altered tumors (55%), 4 *PTEN* (36%), 4 *AR* receptor (36%), 3 *CCND1* (27%), 2 *CDKN2A* (18%), and 2 *FGF family altered tumors* (18%) (Fig. 5).

#### Pancreatic cancer cohort

One patient with pancreatic cancer has been enrolled on this study to date. The patient has biopsy proven metastatic pancreatic adenocarcinoma. GeneTrails analysis revealed genetic alterations in *TP53* and *KRAS.* No actionable genetic alterations were identified in this patient sample.

#### Clinical decision making

In this study, tissue acquisition was designed to optimize conducting multi-omics characterization of tumor samples. At the MM-TERT stage of the SMMART trials initiative, only GeneTrails analytics were CLIA compliant. Therefore, only those results were provided to the treating physician, and to the patient. It was then left to the physician and the patient to decide what, if anything, should be done with the resultant information. Of the 22 subjects with evaluable tumor sequencing results, 5 (23%) patients underwent mutation-guided therapy to date, whose cases are described below. The rest of the patients enrolled on MM-TERT are being followed for clinical outcomes. Additionally, these patients are being considered for subsequent clinical trials based on CLIA assay results from already reported or potential future tissue collections.

#### Patient #1

A 70 year old woman with metastatic hormone-receptor positive breast cancer that developed on adjuvant aromatase inhibitor therapy. She presented with abdominal discomfort, malaise, and diffuse musculoskeletal pain. Patient was started on palbociclib and fulvestrant therapy. Genetrails from metastatic liver biopsy revealed *CDKN2A* and *PIK3CA* alterations. Based on these results, everolimus was added to the treatment plan. Patient tolerated therapy well, and clinically had resolution of her presenting symptoms. She achieved a partial response to therapy lasting 10 months. Patient has since received liposomal doxorubicin for 8 months, and currently on capecitabine therapy. Of note, patient did undergo a repeat liver biopsy on progression, with immunohistochemistry still positive for estrogen receptor, negative for progesterone receptor and HER2. Interestingly, staining was also strongly positive for androgen receptor and BCL-2, based on which she is being considered for enzalutamide or venetoclax based therapies on study.

#### Patient #2

A 38 year old woman with metastatic hormone-receptor positive breast cancer, who has received two lines of endocrine therapy (palbociclib/letrozole and everolimus/exemestane), and two lines of chemotherapy (capecitabine, nab-paclitaxel) in the metastatic setting. GeneTrails analyses from a sternal lesion revealed *CHK2* genetic alteration, *MYC* amplification, and *FANCA* copy number loss. These findings allowed the treating physician to identify a clinical trial combining CHK pathway inhibitors and PARP inhibitors at a different institution, for which the patient was screened. Unfortunately, patient developed rapid hepatic disease progression during screening, and decided to pursue hospice care.

#### Patient #3

A 60 year old man with metastatic castrate-resistant prostate cancer, who has received seven lines of therapy in the metastatic setting, including docetaxel, abiraterone, enzalutamide. GeneTrails analysis revealed *CDKN2A* genetic alteration as well as *WNT* pathway activation. Based on these findings, the patient was placed on combination therapy with a cyclin-dependent kinase 4/6 inhibitor (palbociclib) targeting *CDKN2A* and a selective COX-2 inhibitor (celebrex) targeting the *WNT* pathways [21]. After 2 months of therapy, with PSA trending up (but not meeting progression of disease parameters), the patient elected to switch to cabazitaxel. Patient proceeded to receive two additional lines of standard of care therapy, but unfortunately disease progressed and patient passed away around 6 months after progressing on cabazitaxel therapy.

#### Patient #4

A 68 year old woman with metastatic hormone-receptor positive breast cancer that developed 2 years after completing locoregional therapy for early stage breast cancer. Patient had received letrozole and fulvestrant in the metastatic setting with progression of disease. GeneTrails analysis revealed *CDKN2A* copy number loss, *PIK3CA* pathogenic mutation, and *PTEN* copy number loss. Based on these findings, abemaciclib (CDK 4/6 inhibitor) was added to fulvestrant after progression on single agent fulvestrant, and patient achieved stable disease for 10 months. Based on GeneTrail findings, patient was considered for everolimus based therapy, however she experienced rapid local progression after abemaciclib and fulvestrant. She did undergo repeat biopsy on MM-TERT to evaluate tumor evolution, and then started on single agent capecitabine therapy, which she is currently receiving.

#### Patient #5

A 37 yo woman with metastatic hormone-receptor positive breast cancer, that has received palbociclib/letrozole and everolimus exemestane in the metastatic setting. Patient enrolled on MM-TERT and underwent a liver biopsy which revealed the tumor to be estrogen and progesterone negative, however had become HER2 amplified by immunohistochemistry and GeneTrails. GeneTrails analysis also revealed, among others: *PIK3CA* pathogenic mutation, *MYC* amplification, and regional loss of chromosome 13 (*BRCA2, RB1).* Based on the findings on repeat biopsy, which the patient would not have had under standard of care, she has been receiving HER2 directed therapy with paclitaxel/trasztuzumab/pertuzumab and has achieved a partial response to therapy.

## Discussion

Precision medicine in oncology aims to successfully match genetic alterations within a patient’s tumor with an effective targeted therapy that can subsequently improve survival for that patient. With techniques becoming widely available to sequence tumor DNA, we have witnessed a massive increase in genomic testing in oncology to guide therapy. As an example, Foundation medicine, which provides commercial testing, ran 67,375 tests in 2017. Unfortunately, this exponential adoption of genomic testing has not been paralleled with matching success in the clinic. The implementation of precision medicine in oncology has faced several challenges [14, 15], and to date a minority of patients who undergo testing are successfully treated with a matching therapy. A number of studies have attempted to evaluate the utility of molecular testing based on the number of patients enrolled on clinical trials and the outcomes for these patients. These approaches are important milestones but however fail to account for patients who receive targeted therapy as standard of care such as EGFR inhibitors in lung cancer or BCR/ABL inhibitors in CML. Further they underestimate the utility of these approaches in defining newly emerging therapeutic opportunities such as the recent approval of TRK inhibitors in patients with *TRK* fusion genes.

In this study, we report on the first step in establishing and standardizing a tissue acquisition workflow for a successful real-time spatially resolved, multi-omics driven personalized medicine platform at OHSU, spanning patient consent to molecular tissue analysis reporting. We tailored our approach to overcome previously reported precision medicine limitations. In our patient cohort, we first prove safety and feasibility of obtaining metastatic biopsies without any reported complications. Based on these findings, the OHSU IRB has approved a protocol amendment allowing for obtaining serial biopsies from enrolled patients. To date, four patients receiving standard of care therapy have consented and safely underwent repeat metastatic site biopsies. Second, the majority of obtained biopsies had excellent yield for genomic testing, with only 2 samples reported to have insufficient tumor for analysis. Interestingly, for one of these cases, the patient went on to achieve complete radiographic response, and the other had sclerotic bone tissue on biopsy, indicating response to current therapy, explaining the pathology findings. Third, the GeneTrails custom solid tumor panel identified actionable biology in the large majority of tumors. This is a critical finding as a major hurdle to personalized medicine has been the lack of identified druggable genetic alterations as well as available trials to target the alterations. Last, turnaround time from biopsy to resulting molecular analysis was quick at a median of 17 days. This falls well within the commonly used 28 day washout period in most clinical trials, and thus is unlikely to delay patient care, either on standard regimens or investigational therapies. Moving forward, we have dedicated research staff that (1) work with Knight Diagnostic Laboratories to ensure timely processing and CLIA assay reporting, (2) ensure tissue processing and delivery to the appropriate research labs, and (3) work with clinicians and basic scientists to prioritize and track testing on each collected samples. This will ensure that we are able to deliver on required timelines as we move into the therapeutic clinical trial phase of the SMMART program.

During our implementation of this tissue acquisition and analysis process, we observed a phenomenon that we consider very important and impactful. Specifically, GeneTrails results provided physicians and their patients the opportunity to probe tumor biology, explore novel rational therapies or relevant clinical trials. At the physician level, cancer biologists met with the treating providers to explain the underpinnings of the underlying biology that is represented in the CLIA omic findings. At the patient level, this process is extremely important as it provides new treatment avenues for advanced cancer, where no curative therapies exist to date. Further, it provided patients with a terminal diagnosis an opportunity to explore every possibility, and it did so with a sound rational basis in cancer biology. Of note, MM-TERT enrolled patients at different stages of therapy for metastatic cancer, with a goal to optimize analytic processes. Findings from CLIA assays were provided to treating physicians to act on if deemed appropriate, but the protocol did not mandate a specific therapy. As such, MM-TERT did not compromise patient access to available standard of care therapies. In our study to date, we report on instances where genomic analyses impacted treatment decisions, including developing novel drug combinations, repurposing FDA approved therapies for a different indication, and determining eligibility for investigational clinical trials.

Findings from this study will provide the foundation for the next iteration of the SMMART trials, which will aim to: first, provide patients with rational biologically driven targeted therapies based on proximal biopsies reported in real time to improve their outcomes; second, data generated from the broad analytics will improve our understanding of cancer biology and mechanisms of sensitivity and resistance to therapy; and third, SMMART will look to inform on novel approaches in the field of experimental therapeutics. The next step currently underway is establishing a molecular tumor board comprising medical oncology, molecular pathology, preclinical investigators, computational biology, research coordinators, and clinical pharmacy. The tumor board would meet to review the patient’s clinical, pathologic, and molecular data, inclusive of GeneTrails and a broader suite of tumor-derived analytics, and to cross-reference against best established references to provide recommendations for the next therapy for the patient. Importantly, this is through a process founded in our latest understanding of cancer biology and our latest ability to extract as much information as possible from deep spatially resolved analysis of each individual’s tumor. As serial on-treatment biopsies are allowed per protocol, comparing pre- and post-therapy samples will provide real-time information on tumor biology to identify and target resistance pathways to improve patient outcomes. Further concurrent analysis of circulating tumor DNA will support the utility of that platform in monitoring response and resistance and potentially as a replacement for some tumor biopsies. Finally, with the emergence of immune-oncology, SMMART will include best available biomarkers (including to date PD-L1, microsatellite instability, and tumor mutational burden testing) to identify patients that would potentially benefit from immune therapy combinations.

Another major hurdle to implementing precision medicine has been access to clinical trials, based on location, slot availability, and patient eligibility. In the treatment protocols under the SMMART trials initiative, a list of thirty-three FDA approved therapies will be available to enrolled patients with funding support from philanthropy. These therapies will include chemotherapy, targeted therapies, as well as immune therapies. This truly allows OHSU investigators to implement the molecular tumor board’s recommendations, as it removes a massive obstacle to both clinicians and patients’ attempt to apply personalized medicine. The availability of this therapeutic tool kit, coupled with an analytic process designed to identify “best fit” therapeutic regimens for each patient at each time point in the clinical course of their disease constitutes a design strategy wherein analytics do not exclude patients from entry, but are specifically designed to include all. The comprehensive multi-omics assays embedded in the SMMART platform will serve to identify novel biomarkers, and generate hypothesis for the next generation of clinical trials investigating novel therapy combinations, including novel targeted and immune therapies.

## Conclusions

In conclusion, we report here on our first step towards our personalized medicine platform at OHSU. Using a multidisciplinary approach, we have successfully accrued patients and optimized our workflow in a short period of time. This will now serve as the foundation for the next generation of SMMART trial initiatives aimed at delivering the best therapy to the right patient at the right time, and improve outcomes in advanced cancer.

## Additional file

**Additional file 1: Figure S1.** GeneTrails comprehensive solid tumor panel.** Table S1.** List of Genomic Alterations per MM-TERT Patient.

## Abbreviations

OHSU KCI: Oregon Health and Science University Knight Cancer Institute
MM-TERT: Molecular Mechanisms of Tumor Evolution and Resistance to Therapy
SMMART: Serial Measurements of Molecular and Architectural Responses to Therapy
