# Supplementary material for: Implementing a comprehensive translational oncology platform: from molecular testing to actionability
Source: J Transl Med. 2018 Dec 14;16:358. doi: 10.1186/s12967-018-1733-y (PMC6295039; doi:10.1186/s12967-018-1733-y)
Supplement: Supplementary file 1 — Additional file 1: Figure S1. GeneTrails comprehensive solid tumor panel. Table S1. List of Genomic Alterations per MM-TERT Patient. [file 12967_2018_1733_MOESM1_ESM.pdf]

Figure S1. GeneTrails Comprehensive Solid Tumor Panel

AKT1	CDKN1B	FANCM	KIT	NTRK1	RAD51D
AKT2	CDKN2A	FGF18	KRAS	NTRK2	RAD52
AKT3	CHEK1	FGF19	MAP2K1	NTRK3	RAD54L
ALK	CHEK2	FGF3	MAP2K2	PALB2	RAF1
APC	CTNNB1	FGF4	MAP2K4	PDCD1LG2	RASA1
AR	DDR2	FGFR1	MAPK1	PDGFRA	RB1
ARAF	DDX11	FGFR2	MDC1	PIK3CA	RET
ARID1A	EGFR	FGFR3	MDM2	PIK3CB	RICTOR
ATM	ERBB2	FGFR4	MDM4	PIK3R1	RIT1
ATR	ERBB3	GNA11	MET	PMS1	ROS1
BAP1	ERBB4	GNAQ	MLH1	PMS2	RPTOR
BARD1	ERCC2	GNAS	MLH3	POLE	STAG2
BRAF	ERCC5	HIST1H3B	MRE11A	PPP2R1A	STAT3
BRCA1	ESR1	HRAS	MSH2	PPP6C	STK11
BRCA2	FAM175A	IDH1	MSH6	PTCH1	TOP1
BRIP1	FANCA	IDH2	MTOR	PTEN	TP53

Table S1. List of Genomic Alterations per MM-TERT Patient

**101**	PIK3CA p.E542K (~43% mutant allele)<br>CDKN2A gene copy number loss (~1.0 copy)<br>ATM gene copy number loss (~0.5 copies)<br>Chr 11 regional amplification, including CCND1, FGF19, FGF3, and FGF4 (~9-30 copies - CCND1 highest)<br>Chr 1 regional amplification, including MDM4 and DDR2 (~6-7 copies)<br>Chr 11 regional loss, including CHEK1 and MRE11 (~0.6-0.7 copies)<br>Chr 8 regional loss, including FGFR1, IDO1 and IDO2 (~0.5 copies each)<br>Chr 17 regional loss, including RAD51D (~1.0 copy)
**104**	PIK3CA p.E542K<br>CDKN2A copy number loss<br>Chr11 regional amplification (CCND1 and FGF19)
**105**	PIK3CA p.E542K<br>PTEN copy number loss<br>TP53 copy number loss<br>CDKN2A copy number loss
**106**	PIK3CA p.C420R and copy number gain<br>TP53 p.P250L
**109**	CDKN2A copy number loss
**110**	CHEK2 p.T367fs*15<br>MYC copy number gain<br>FANCA copy number loss
**111**	PIK3CA p.H1047R<br>TP53 p.E285<br>ERBB3 copy number gain<br>FGF19 copy number gain
**113**	PTEN p.T319fs*1 and copy number loss
**114**	RAD51D p.S207L<br>FGFR2 p.N550K<br>PIK3CA p.E542K<br>mTOR p.M2089I, p.I1094M and p.D907H<br>PTEN splice site mutation and p.P248fs*2<br>STK11 p.S69*<br>PIK3CA p.P104_G106delinsR<br>CASP8 p.S403*<br>ARID1A p.Q1424*

**115**	PTEN gene copy number loss<br>CCND1 gene copy number gain (8 copies)<br>CDKN2A copy number loss (0 copies)<br>Chr 8 regional amplification, including FGFR1 (23 copies), IDO2 (16 copies), IDO1 (8 copies) and MYC (5 copies)
**116**	CDKN2A copy number loss (0.8 copies)<br>BRCA2 copy number loss (0.8 copies)
**117**	TP53 p.R248W
**201**	TP53 copy loss<br>TMPRSS2-ERG gene fusion
**204**	PTEN p.C71fs*30<br>ATR p.I2164M
**207**	CTNNB1 p.T41A<br>CDKN2A copy number loss<br>Chr 11 regional amplification, including CCND1, FGF19 and MRE11A
**208**	AR gene amplification<br>MYC copy number increase<br>CHEK1 copy number loss<br>ATM copy number loss<br>Chr 11 regional gain, including CCND1, FGF19, FGF3, and FGF4
**211**	PTEN p.S170fs*13<br>RB1 copy number loss
**212**	ERCC2 splice site mutation<br>AR gene amplification<br>ATM splice site mutation and copy loss
**213**	CDK12 p.R221fs*6<br>CDKN2A gene copy number loss (0.2 copies)<br>ERCC5 gene copy number loss (0.2 copies)<br>TMPRSS2-ERG gene fusion
**214**	ATM p.I1476fs*15<br>PTEN p.T277fs*21<br>ERCC5 p.S470*<br>Chr 11 regional gain, including CCND1, FGF19, FGF4 and FGF3 genes (~9-15 copies)
**215**	RAD50 p.D1294N<br>TP53 p.K132N<br>AR gene amplification<br>TMPRSS2-ERG gene fusion

**218**	TP53 p.R175H<br>AR gene amplification<br>PTEN copy number loss<br>TMPRSS2-ERG gene fusion
**301**	KRAS p.G12D<br>TP53 p.P151H
